# Supplementary material for: A Survey of Patients’ Opinions and Preferences on the Use of E-Prescriptions in Poland
Source: Int J Environ Res Public Health. 2021 Sep 16;18(18):9769. doi: 10.3390/ijerph18189769 (PMC8467067; doi:10.3390/ijerph18189769)
Supplement: Supplementary file 1 [file ijerph-18-09769-s001.zip › ijerph-1365090-supplementary.pdf]

The questionnaire used in the study

Gender:	Age:	Education:	Place of residence:
☐ Female	☐ less than 30 years	☐ primary	☐ Village
☐ Male	☐ 31-50 years	☐ junior high school	☐ Small town *(<20000 residents)*
	☐ 51-70 years	☐ secondary	☐ Medium-sized town *(20-100 thousand residents)*
	☐ above 70 years	☐ vocational	☐ Big city *(>100 thousand residents)*
		☐ higher	

Question 1

Are you chronically ill with any diseases?

☐ Yes ☐ No

Question 2

How have you used your medications in the last 6 months?
(you can choose only one answer)

- ☐ Mostly chronic
  (using the same drugs for a long time for chronic diseases, e.g. diabetes, hypertension, etc.)
- ☐ Mostly ad hoc
  (use of drugs only in temporary diseases, e.g. infections, injuries, etc.)
- ☐ Both chronically and temporarily

Question 3. (you can choose only one answer)

How often in the last 6 months have you filled your prescription at a pharmacy?

☐ never ☐ once-twice ☐ 3-4 times ☐ 5-6 times ☐ more than 6 times

Question 4.

When you get a prescription from a doctor, how many drugs are prescribed on average?

☐ 1 ☐ 2-5 ☐ 6-10 ☐ more than 10

Question 5

In the last 6 months, have you asked someone else to fill your prescription?

☐ yes ☐ no

<u>Question 6.</u>

In the last 6 months, how often have you filled a prescription for someone else?

☐ never ☐ once-twice ☐ 3-4 times ☐ 5-6 times ☐ More than 6 times

<u>Question 7</u> *(if you answered "never" to the previous question, you can skip this question)*

When you fill prescriptions for someone else, for whom are the medications prescribed?
(*you can choose several answers)*

☐ Partner/Spouse ☐ Child ☐ Parent ☐ Other person (who?) ………………………..

<u>Question 8</u>

Do you know what an electronic prescription (e-prescription) is?

☐ yes ☐ no

If you answered "no" to this question, you can go to ***question 13***

<u>Question 9</u>

Have you dispensed an e-prescription at a pharmacy (for yourself or another person) in the last 6 months

☐ yes ☐ no

<u>Question 10</u>

If you had a choice, which prescriptions would you prefer to receive?
(you can choose only one answer)

☐ paper-based,
Please justify your answer
……….

☐ eletronic,
Please justify your answer
……….

☐ It doesn't matter to me

Question 11

Please, respond to the following statements by ticking the appropriate answer.
(you can only mark one answer per line)

1- strongly disagree<br>2- rather disagree<br>3- no opinion<br>4- rather agree<br>5- strongly agree	strongly disagree	rather disagree	no opinion	rather agree	Strongly agree
	1	2	3	4	5
I think that e-prescriptions increase my autonomy during the therapy					
With e-prescriptions I have a greater control over the prescribed drugs					
I think that e-prescriptions are eco-friendly					
I think that e-prescriptions enhance the access to drugs					
With e-prescriptions it is easier to buy drugs for another person					
I have concerns over my privacy when using e-prescriptions					

Question 12 *(you can choose only one answer)*

On a scale of 1 to 5, how would you rate your overall satisfaction with using an e-prescription?

1 means „very bad”, 5 means „very good”

1	2	3	4	5

Question 13

In what form do you usually receive a prescription from a doctor
(you can choose only one answer)

- ☐ Information printout with the barcode
- ☐ SMS
- ☐ e-mail
- ☐ I receive paper-based prescription
- ☐ other (what?)……………….

Question 14

What form of prescription is the most convenient for you? (you can choose only one answer)

- ☐ Information printout with the barcode
- ☐ SMS
- ☐ e-mail
- ☐ paper-based prescription

Question 15

Do you know what Online Patient Account (OPA) is?

☐ yes ☐ no

Question 16

Do you use Online Patient Account (OPA)?

☐ yes ☐ no

Question 17 (If you answered yes to the previous question 16)

Please, respond to the following statements by ticking the appropriate answer.
(you can only mark one answer per line)

1- strongly disagree
2- rather disagree
3- no opinion
4- rather agree
5- strongly agree

	Strongly disagree	Rather disagree	No opinion	Rather agree	Strongly agree
	1	2	3	4	5
Logging in to the Online Patient Account is easy					
The Online Patient Account service is legible and understandable					
I can easily check the status of all prescriptions issued to me					
OPA always works without problems					
